# Assessing the Quality of Tuberculosis Evaluation for Children with Prolonged Cough Presenting to Routine Community Health Care Settings in Rural Uganda

**DOI:** 10.1371/journal.pone.0105935

**Published:** 2014-08-29

**Authors:** Carina Marquez, J. Lucian Davis, Achilles Katamba, Priscilla Haguma, Emmanuel Ochom, Irene Ayakaka, Gabriel Chamie, Grant Dorsey, Moses R. Kamya, Edwin Charlebois, Diane V. Havlir, Adithya Cattamanchi

**Affiliations:** 1 Division of HIV/AIDS, San Francisco General Hospital, University of California San Francisco, San Francisco, California, United States of America; 2 Division of Pulmonary and Critical Care Medicine and Curry International Tuberculosis Center, San Francisco General Hospital, University of California San Francisco, San Francisco, California, United States of America; 3 School of Medicine, Makerere University College of Health Sciences, Kampala, Uganda; 4 MU-UCSF Research Collaboration, Kampala, Uganda; 5 Division of Infectious Diseases, San Francisco General Hospital, University of California San Francisco, San Francisco, California, United States of America; 6 Center for AIDS Prevention Studies, Department of Medicine, University of California San Francisco, San Francisco, California, United States of America; Fundació Institut d'Investigació en Ciències de la Salut Germans Trias i Pujol, Universitat Autònoma de Barcelona, CIBERES, Spain

## Abstract

**Background:**

Improving childhood tuberculosis (TB) evaluation and care is a global priority, but data on performance at community health centers in TB endemic regions are sparse.

**Objective:**

To describe the current practices and quality of TB evaluation for children with cough ≥2 weeks' duration presenting to community health centers in Uganda.

**Methods:**

Cross-sectional analysis of children (<15 years) receiving care at five Level IV community health centers in rural Uganda for any reason between 2009–2012. Quality of TB care was assessed using indicators derived from the International Standards of Tuberculosis Care (ISTC).

**Results:**

From 2009–2012, 1713 of 187,601 (0.9%, 95% CI: 0.4–1.4%) children presenting to community health centers had cough ≥ 2 weeks' duration. Of those children, only 299 (17.5%, 95% CI: 15.7–19.3%) were referred for sputum microscopy, but 251 (84%, 95% CI: 79.8–88.1%) completed sputum examination if referred. The yield of sputum microscopy was only 3.6% (95% CI: 1.3–5.9%), and only 55.6% (95% CI: 21.2–86.3%) of children with acid-fast bacilli positive sputum were started on treatment. Children under age 5 were less likely to be referred for sputum examination and to receive care in accordance with ISTC. The proportion of children evaluated in accordance with ISTC increased over time (4.6% in 2009 to 27.9% in 2012, p = 0.03), though this did not result in increased case-detection.

**Conclusion:**

The quality of TB evaluation was poor for children with cough ≥2 weeks' duration presenting for health care. Referrals for sputum smear microscopy and linkage to TB treatment were key gaps in the TB evaluation process, especially for children under the age of five.

## Introduction

The World Health Organization (WHO) estimates that over half a million children develop active tuberculosis (TB) and 70,000 children die from TB each year [1]. Despite this large estimated burden of childhood TB, the epidemic has historically been neglected and childhood cases are under-diagnosed and under-reported [2]–[4]. To turn the tide on this hidden epidemic the WHO issued the Roadmap for Childhood TB: Towards Zero Deaths [5]. A central feature of the WHO roadmap is its emphasis on strengthening the capacity of community health systems to diagnose and treat childhood TB. Community health centers are often the first place children with TB interface with the health care system. However, there are sparse data from such settings on whether children who should be evaluated for TB begin or complete the TB evaluation and care cascade.

The International Standards of Tuberculosis Care (ISTC) [6] are evidence-based performance standards that community health centers and national TB programs can use to assess the quality of their current practices related to TB evaluation and care. In adults receiving care at 5 primary health clinics in Uganda, we previously reported low baseline adherence to ISTC-based quality metrics. However, we found that adherence to ISTC-recommended TB evaluation practices and case detection increased during the implementation of a monitoring and evaluation system [7]. To our knowledge, there are no published data from primary health centers in East Africa on the quality of pediatric TB evaluation.

In this report we describe childhood TB evaluation at 5 community clinics in rural Uganda using detailed quality metrics linked to the ISTC for one entry point into the TB diagnosis and care cascade: presentation to a community clinic with cough lasting for two weeks or more. In addition, we sought to characterize community-based practices for management of children with prolonged cough.

## Methods

### Study setting and participants

All children (age <15 years) presenting to five Uganda Ministry of Health Level IV Health Centers for evaluation for any reason between January 2009 and December 2012 contributed data to this cross-sectional analysis. The 5 health centers are geographically dispersed throughout Uganda, with one each in the eastern, northern and central regions and two in the western region. Each health center provides primary care, obstetric, and basic surgical services free-of-charge to a catchment population of approximately 100,000 people. Each has a laboratory that provides basic diagnostic services including sputum acid-fast bacilli (AFB) smear microscopy. Chest radiography, tuberculin skin testing, sputum induction, and gastric lavage are not available on-site but clinicians can refer patients to the district hospital for additional diagnostic testing or care.

We chose the five health centers based on their participation in an infectious disease surveillance network that started with a focus on malaria in 2001 and expanded to include TB in 2009 [8]. The surveillance network captures demographic and clinical information on every patient encounter through a one-page data collection form. Clinical information includes symptoms (fever and cough ≥2 weeks' duration), laboratory tests ordered, laboratory test results, clinician diagnoses, drug prescriptions, and referrals to a higher level of care. To ensure the completeness of TB-related data collection, laboratory data and treatment information are crosschecked with TB Laboratory and Treatment registers.

### Measurements and Definitions

We considered cough ≥2 weeks' duration as a minimum initial entry point for TB evaluation, as recommended by the ISTC [6]. We then evaluated three quality metrics that reflect ISTC-recommended TB evaluation practices: (1) proportion of children with cough ≥2 weeks referred for sputum examination; (2) proportion of children completing sputum examination if referred, defined as having at least one positive or two negative sputum smears examined; (3) proportion of children initiated on TB treatment if sputum AFB smear-positive. We also assessed one summary metric; the proportion of children with cough ≥2 weeks who were evaluated in accordance with all three ISTC-recommended TB evaluation practices.

### Statistical Methods

We used the chi-squared test to compare proportions and the Wilcoxon rank-sum test to compare medians. For quality indicators, we used logistic regression models with robust standard errors to account for clustering to obtain point estimates and 95% confidence intervals (CI), stratified by year. We used the chi-squared test for heterogeneity and trend to evaluate changes in quality indicators over time. We calculated risk ratios (RR) and their 95% confidence intervals (CIs) for the association between age and selected quality indicators using a Poisson model with a log link and robust standard errors, adjusting for calendar year and clinic site. Analyses were performed using STATA 12 (Stata Corporation, College Station, TX).

### Human Subjects

The Makerere University School of Medicine Research and Ethics Committee, the University of California, San Francisco Committee on Human Research, and the Uganda National Council for Science and Technology approved the electronic surveillance system and waived the requirement for informed consent.

## Results

There were 193,177 clinic encounters for children <15 years old during the study period, and children under age 5 comprised 56% of these visits. The median age was 3.7 years (IQR 1.3–9.0) and 53% of children were female. The most common diagnosis for all visits was malaria (41.3%, 95% CI: 41.1–41.5%), followed by upper respiratory infection (36%, 95% CI: 35.8–36.2), diarrhea (8.2%, 95% CI: 8.1–8.3%), and pneumonia (2.4%, 95% CI: 2.4–2.5%).

### Screening for prolonged cough

**Figure 1:**
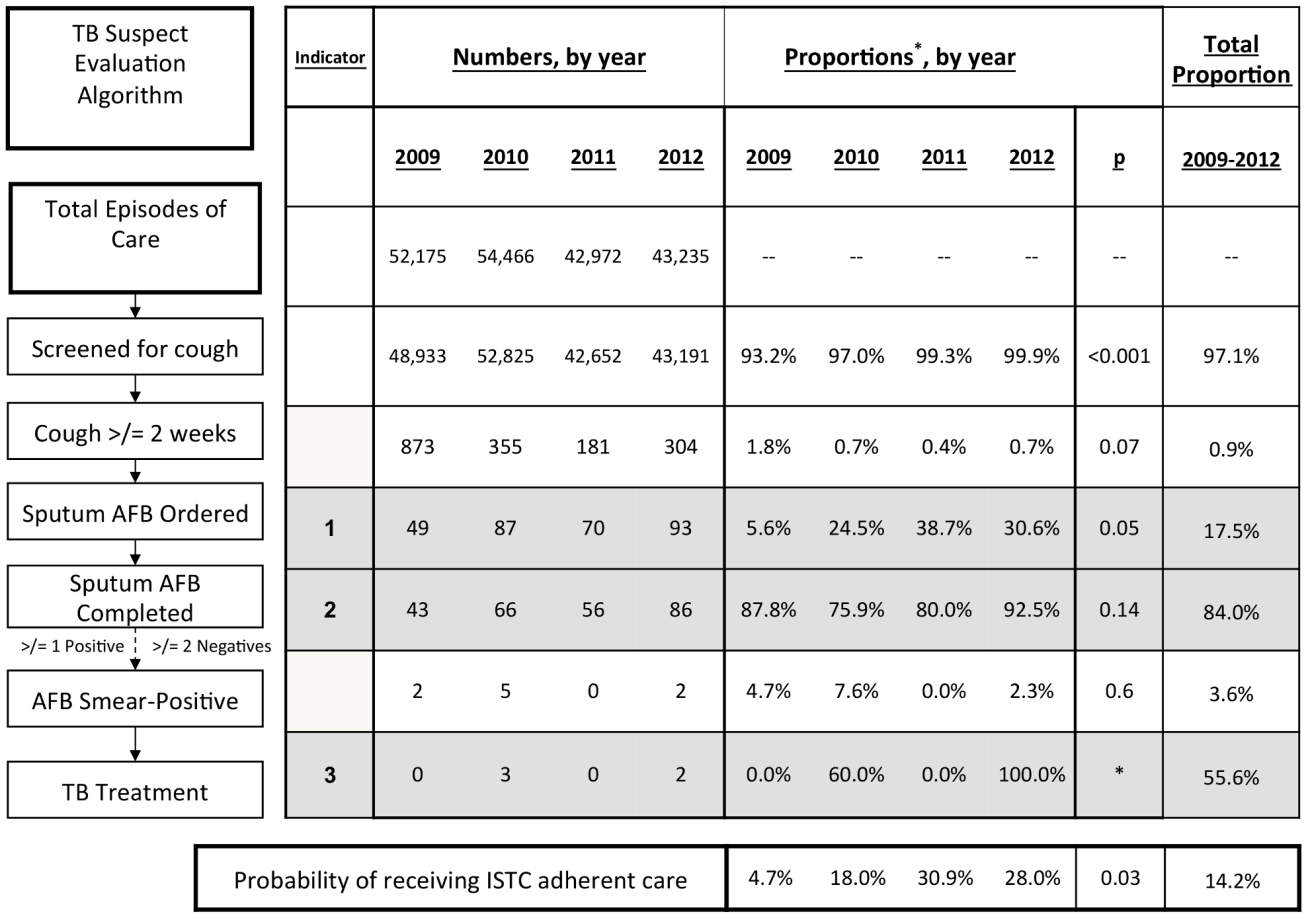
Diagnoses and respiratory antibiotic treatment given to children with cough ≥2 weeks' duration from 2009–2012. A. Proportion of children with cough ≥2 weeks' duration given a primary diagnosis of upper respiratory infection (URI), malaria, pneumonia, bronchitis, or asthma. B. Proportion of children with cough ≥2 weeks' duration receiving respiratory antibiotics, stratified by clinical diagnosis.

**Figure 2:**
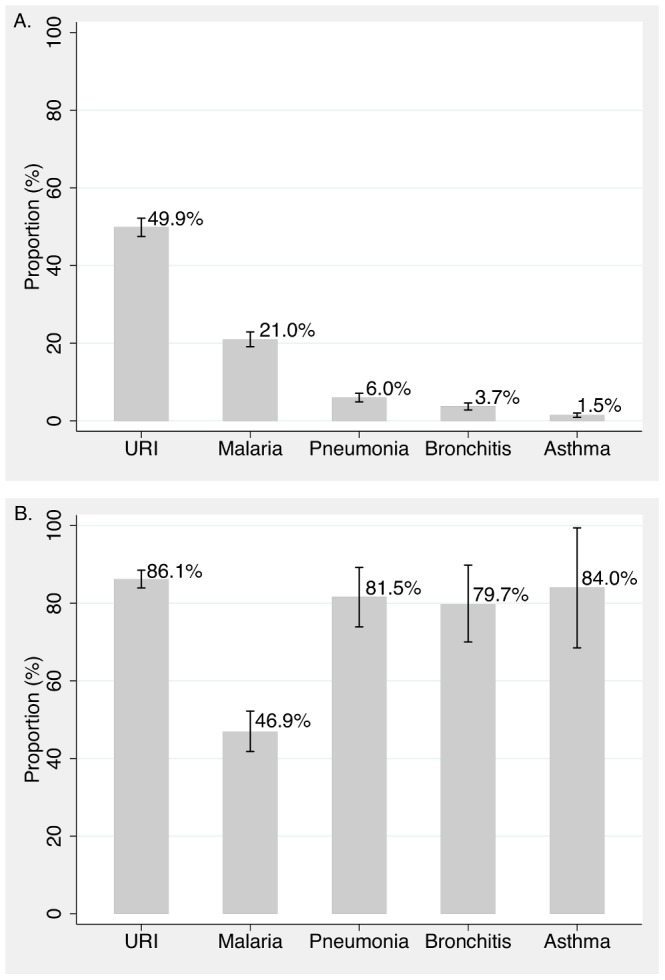
Performance Indicators for children ages 0–15 with tuberculosis symptoms, 2009–2012. Adherence to performance indicators presented as absolute numbers and yearly proportions. Overall, from 2009–2012, 17.5% of children with cough ≥2 weeks' duration were referred for sputum microscopy. Of the children referred for sputum microscopy, 84.0% completed microscopy, 3.6% had AFB-positive sputum, and 55.6% were started on TB treatment. From 2009–2012, there were marginal increases in the number of children referred for sputum microscopy, but this did not result in an increase in TB case-detection. *Proportions account for clustering by site and differ from the proportion calculated by the raw numbers. **Proportion of children who cough ≥2 weeks' duration who received all ISTC-recommended care is defined as the probability that a child presenting with cough will have sputum microscopy ordered, complete sputum examination, and start TB treatment if AFB smear-positive.

During the study period 187,601 (97.1%, 95% CI: 93.8–100%) children were screened for having cough ≥2 weeks' duration. The proportion screened was high throughout the study period, ranging from 93.2% in 2009 to 99.9% in 2012. Among those screened, 1,713 (0.9%, 95% CI: 0.4–1.4%) children reported having prolonged cough, with prevalence ranging from 0.4% to 1.3% across sites. The proportion of children with prolonged cough did not vary by calendar year (Figure 1). The most common diagnoses for children with prolonged cough were upper respiratory tract infection (URI) (49.9%, 95% CI: 47.5–52.2%), followed by malaria (21.0%, 95% CI: 19.1–22.9%) and pneumonia (6.0%, 95% CI: 4.9–7.1%) (Figure 2A). Respiratory antibiotics (trimepthoprim-sulfamethoxazole, penicillin, amoxicillin, ceftriaxone, erythromycin, or doxycycline) were prescribed for 66.6% (95% CI: 64.4–68.8%) of children with prolonged cough and for 77.6% (95% CI: 72.8–82.3%) of those referred for sputum AFB examination. Stratified by diagnoses, of those children with prolonged cough, respiratory antibiotics were prescribed for 86.1% of children with a URI, 46.9% with malaria, 81.5% with pneumonia, 84.0% with asthma, and 79.7% with bronchitis (Figure 2B). There was no difference in prescribing practices between respiratory illnesses, p = 0.504. Antibiotic prescriptions were high regardless of cough duration, among children with and without a prolonged cough, 82.0% with URI, 32.7% with malaria, 85.5% with pneumonia, 77.6% with asthma, and 85.1% with bronchitis received a respiratory antibiotic.

### The Quality of TB Evaluation and Linkage to Care

#### Referral for sputum examination

The proportion of children with prolonged cough referred for sputum AFB examination was 17.5% (95% CI: 15.7–19.3%). The proportion referred for sputum AFB examination increased from 5.6% (95%CI: 0.10–11.2%) in 2009 to 24.5% (95%CI: 5.7–43.3%) in 2010 to 38.7%(95% CI: 0–80.2%) in 2011, before leveling off at 30.6% (5.1–56.0%) in 2012 (p≤0.05, chi-squared test for trend) (Figure 1).

#### Completion of sputum examination

The proportion of children who completed sputum examination if referred was 84.0% (95% CI: 79.8–88.1%). The proportion ranged from a low of 75.9% (95% CI: 64.2–87.5%) in 2010 to a high of 92.5% (95%CI: 85.0–99.9%) in 2012 (p = 0.14, chi-squared test for trend from 2009–2012) (Figure 1).

#### Treatment initiation and case detection

Of the 251 children who completed sputum microscopy, 9 were AFB smear-positive (3.6%, 95% CI: 1.4–5.9%) and 55.6% (95% CI: 21.2–86.3%) of the children with AFB smear-positive sputum were started on TB treatment. The small number of AFB smear-positive children precluded trend analysis; however, the absolute numbers of children who were initiated on TB treatment if AFB smear-positive were low: 0 of 2 in 2009, 3 of 5 in 2010, 0 of 0 in 2011, and 2 of 2 100% in 2012 (Figure 1).

#### Proportion receiving ISTC-adherent care

The proportion of children evaluated in accordance with ISTC recommendations was 14.2% (95% CI: 12.7–16.0%) (Figure 2). ISTC adherence increased over time from 4.7% (95% CI: 0.10–9.3%) in 2009 to 28% (95%CI: 2.9–53.1%) in 2012 (p = 0.03, chi-squared test for trend from 2009–2012). Of note, only 0.53% (95%CI: 0.18–0.87%) of children with prolonged cough were referred to a higher level of care for further evaluation and only one child was referred for chest radiography.

### Age- and Gender-Related Differences in the Quality of TB Evaluation

Children over the age of 5 were more likely to be referred for AFB microscopy and to receive ISTC-adherent care (Table 1), when adjusting by site and year. Only 5.0% (95% CI: 0.0–10.8%) of children under age five with prolonged cough were referred for sputum AFB examination compared to 28.0% (95% CI: 23.1–42.9%, RR 4.1, p = 0.01) of children 5–9 years and 28.6% (95% CI: 4.8–53.4%, RR 4.73, p = 0.02) of children 10–15 years. However, a similar proportion of children across all age groups completed sputum examination if referred. There were too few children with positive AFB smears to evaluate age-specific differences in treatment initiation. There were no gender-related differences in any of the quality indicators.

**Table 1:** Age-specific analysis of selected quality indicators.

	Referral for Sputum Microscopy		Completed Sputum Microscopy		Received ISTC-adherent care	
Age (years)	RR (95% CI)	p	RR (95% CI)	p	RR (95% CI)	p
<5	1		1		1	
5–9	4.6 (1.4–15.3)	0.013	1.2 (1.0–1.4)	0.108	5.2 (3.5–7.8)	<0.001
10–15	4.7 (1.3–17.7)	0.021	1.2 (1.0–1.4)	0.138	5.3 (3.5–8.0)	<0.001

Reference group 0–5 years.

Models adjusted by site, and year of clinical encounter.

### Between-Site Differences in the Quality of TB Evaluation

Yearly adherence to quality indicators varied significantly by clinic, and one site did not exhibit improvement in any indicators. Averaged over the study period individual clinic adherence to quality indicators ranged from: 7.4–42.5% for referral to sputum examination, 70.9–95.9% for completion of sputum examination if ordered, and 7.0–37.7% for evaluation in accordance with the ISTC. There were too few smear-positive TB cases to assess site-level differences in treatment initiation.

## Discussion

In this study, we describe the quality of routine TB diagnostic services for children with cough ≥2 weeks' duration attending community health clinics in rural Uganda. We found adherence to ISTC-recommended TB evaluation practices to be low, especially for children under five. The primary breakdown in the diagnostic pathway was referral for sputum smear microscopy: from 2009–2012, only 18% of children with prolonged cough were referred for sputum smear microscopy and only 0.53% were referred to a higher level of care for further evaluation. Case detection was low throughout the study period, and did not increase even with improved ISTC adherence from 5% in 2009 to 28% in 2012. Our findings highlight the need for interventions to increase testing of children for TB and for increased uptake of clinical algorithms for TB diagnosis.

To our knowledge, this study is the first of its kind to prospectively describe childhood TB evaluation practices in a resource-constrained, community-based setting using well-defined quality metrics. In Pakistan, Safdar *et al.* conducted retrospective reviews of children diagnosed with TB in district hospitals and found poor baseline adherence to National TB Program-recommended diagnostic practices, which included tuberculin skin testing, chest x-rays, and clinical scoring systems [9], [10]. A retrospective study in Malawi also found poor adherence to diagnostic guidelines in children hospitalized with TB [11]. Our study examines earlier steps in the diagnostic cascade through analysis of outpatient visits of children with prolonged cough, and highlights referral for TB testing as an important gap in the quality of evaluation.

Children may have difficulty expectorating sputum [2], [12], and this may be one reason health care workers may fail to refer children for sputum examination. However, we found that over 80% of children referred for sputum microscopy were able to complete smear examination, and the probability of completing the test did not change when the proportion of children referred for microscopy increased from 6% in 2009 to 31% in 2013. Although not all children can produce sputum, these data suggest that the test is not being ordered in many that could have produced sputum. Additionally, for those children who are unable to expectorate sputum, sputum induction has been shown to be feasible and to increase case detection in resource-limited primary health care settings [13], and may be one way to improve the clinic's capacity to obtain sputum and adhere to ISTC standards.

The marginal improvement in ISTC-adherent care from 5% in 2009 to 28% in 2012 was not associated with an increase in the number of childhood TB cases detected, which remained low, in contrast to our previous finding in adults [7]. Pediatric TB case detection in community settings is rare [14], [15] and although sputum microscopy is the first-step in TB evaluation, pediatric TB is often smear-negative. Therefore, larger improvements in ISTC-adherent care may be needed to identify more smear-positive TB cases and would also maximize the impact of the ongoing scale-up of the Xpert MTB/RIF assay. The 2–3 fold higher sensitivity of Xpert MTB/RIF relative to smear microscopy [16] will lead to meaningful increases in case detection only if children with TB symptoms and signs are identified and referred to testing. However, because even Xpert MTB/RIF has sub-optimal sensitivity among children and sputum collection may not be feasible in community settings, improving the quality of TB evaluation should also focus on training providers to make a diagnosis of childhood TB on clinical grounds. The Union Desk Guide for Diagnosis and Management of TB in Children provides a simple, feasible, and evidence-based clinical algorithm for identification of childhood TB cases [17]. Further studies are needed on strategies to promote uptake of the Desk Guide, and that evaluate its impact.

In addition to baseline descriptive data on childhood TB evaluation, our study also identified other potential areas for improvement in pediatric lung health. Among children with cough, regardless of duration, we found overprescribing of antibiotics (over 80% of children diagnosed with a upper respiratory infection received antibiotics) and possible under-treatment of pneumonia (an antibiotic prescription was not recorded in 18% of children diagnosed with pneumonia). Pneumonia and acute respiratory infections are among the leading causes of death in children worldwide [18], and integrated quality improvement and case management, with tools such as WHO's Practical Approach to Lung Health, should be considered to improve general pediatric lung health [19].

This study has several potential limitations. The monitoring and evaluation system may underestimate the quality of service provided if data are captured incompletely; however, we mitigated this source of bias by cross-checking our data with existing TB laboratory and treatment registers at the sites. Additionally, this study only presents a diagnostic pathway for one TB symptom and does not address the quality of evaluation of children presenting with other TB symptoms or a history of contact to a TB case, which are critical elements of pediatric TB evaluation. However, there were few childhood TB diagnoses among children without cough, suggesting that our study did not miss another pathway by which childhood TB is being diagnosed.

In summary, measurement of adherence to TB quality metrics revealed poor quality of care and a low number of TB diagnoses among children presenting to primary health centers. There is a need for improved understanding of the provider and clinic-level factors that impact adherence to recommended TB evaluation practices in order to promote screening, testing and treatment of TB among children. Such strategies are critical for ensuring that children interfacing with community health care settings do not fall off the TB diagnosis and treatment care cascade.
